# Effect of enriched environment on postoperative sleep and recovery quality in patients undergoing laparoscopic surgery for colorectal cancer

**DOI:** 10.3389/fonc.2026.1627839

**Published:** 2026-04-10

**Authors:** Pengfei Zhang, Dongzhi Jiang, Huiwei Deng

**Affiliations:** Department of Anesthesiology, Changde Hospital, Xiangya School of Medicine, Central South University (The First People’s Hospital of Changde City), Changde, Hunan, China

**Keywords:** colorectal cancer, laparoscopic surgery, multimodal interventions, postoperative recovery quality, postoperative sleep quality

## Abstract

**Objective:**

To explore the effect of a multimodal interventions (MI) on postoperative sleep and recovery quality in patients undergoing laparoscopic surgery for colorectal cancer (CRC).

**Methods:**

266 patients with CRC undergoing laparoscopic surgery were randomly divided into MI (intervention group) and control (control group) groups. Postoperative treatment in control group was carried out according to medical standards, while MI was provided based on routine treatment in intervention group. The sleep at night before surgery (N_-2_) after surgery (N_0_), and 6th night after surgery (N_6_) were collected. The Pittsburgh Sleep Quality Index (PSQI) and 15 Recovery Quality Inventory (QoR-15) scores on the 1st day before surgery (D_-1_) and after surgery (D_1_), and the 7th day after surgery (D_7_) were collected. The postoperative complications and NRS pain scores from days 1-3 (D_1-3_) were collected.

**Results:**

At N_6_, patients in control group had increased awake time, decreased sleep time, and decreased sleep efficiency after falling asleep than intervention group (*P* < 0.05). Compared with D_-1_, both groups of patients showed an increase in PSQI scores at D_1_ and D_7_, while a decrease in QoR-15 scores (*P* < 0.05). Compared with control group, intervention group had a shorter latency period for falling asleep at N_0_ (*P* < 0.05). At D_1_ and D_7_, the PSQI score was lower and the QoR-15 score was higher (*P* < 0.05). At D_1_ and D_2_, the NRS pain score during exercise was lower and satisfaction was higher (*P* < 0.05).

**Conclusion:**

In patients with laparoscopic surgery for colorectal cancer, MI can improve postoperative sleep, recovery quality, and postoperative recovery.

## 1 Introduction

Colorectal cancer (CRC) ranks as the third most prevalent malignancy globally and remains a leading cause of cancer-related mortality (1). In China, an estimated 555,000 new CRC cases were reported in 2020, making it the second most common cancer, with 286,000 associated deaths, ranking fifth in cancer-related mortality (2). The incidence and mortality of CRC continue to rise in China and other developing countries (3, 4), with an increasing proportion of younger patients presenting at advanced stages. Among patients under 50 years of age, 26% are diagnosed with distant metastases, compared to 23% and 19% in the 50–64 and ≥65 age groups, respectively (1). Despite accounting for 31% of global CRC cases, early-stage detection rates in China remain low (5, 6).

Surgical resection combined with appropriate perioperative care remains a cornerstone of CRC management (7). Advances in surgical techniques, anesthesia, and perioperative management have significantly reduced operative risks (8). Nevertheless, postoperative patients frequently experience pain, nausea, vomiting, reduced oral intake, and limited mobility, all of which contribute to postoperative sleep disturbances (PSD) (9). PSD, though common, has historically received insufficient attention despite its critical role in patient recovery. Sleep disruption negatively impacts immune function, pain perception, metabolic homeostasis, and overall physical and psychological rehabilitation, potentially delaying recovery and prolonging hospital stay (10). In severe cases, PSD and related complications may even lead patients to refuse necessary surgical interventions. Therefore, strategies aimed at mitigating postoperative sleep disturbances are essential for optimizing recovery, enhancing patient satisfaction, and improving long-term outcomes.

Multimodal interventions (MI), initially conceptualized by Hebb, encompass therapeutic approaches that integrate sensory stimuli such as sound, light, and color to enhance environmental enrichment, cognitive engagement, social interaction, physical activity, and spatial exploration (11, 12). MI offers notable advantages, including low cost, ease of implementation, and high adaptability, making it a practical non-pharmacological strategy. Within the biopsychosocial framework of modern medicine, MI represents a promising avenue for addressing postoperative sleep disturbances and facilitating recovery (13, 14). However, evidence regarding the efficacy of MI specifically in patients undergoing laparoscopic CRC surgery remains limited. Accordingly, the present study aimed to evaluate the effect of MI on postoperative sleep quality and overall recovery in this patient population.

## 2 Subjects and methods

### 2.1 Subjects

This prospective randomized controlled study was approved by the Ethics Committee of the First People’s Hospital of Changde City (Approval No. YX-2023-026-02). Written informed consent was obtained from all participants or their authorized representatives.

**Figure 1:**
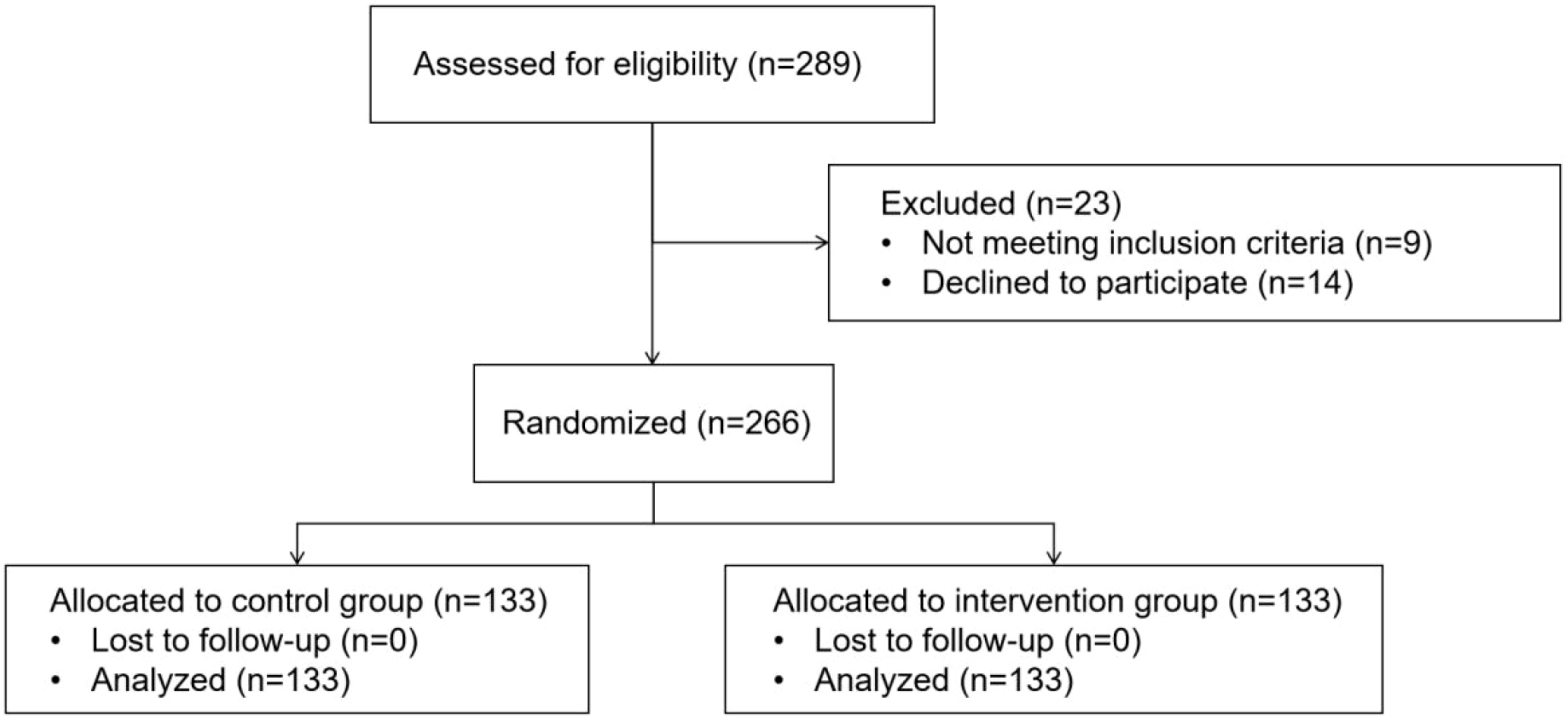
Participant flow diagram.

A total of 266 patients with pathologically confirmed colorectal cancer (CRC) who underwent elective laparoscopic surgery at our institution between April 2023 and October 2024 were enrolled (**Figure 1**). Eligible patients were aged 18–65 years, had a body mass index (BMI) of 18.5–28 kg/m², were classified as American Society of Anesthesiologists (ASA) physical status I–III, underwent laparoscopic radical CRC resection under general anesthesia, and had an expected operative duration of 2–4 hours.

Exclusion criteria included allergy to anesthetics or long-term sedative/analgesic use, insomnia treated with hypnotics within the previous month, severe cardiac, pulmonary, hepatic, renal, or psychiatric disease, marked intraoperative hemodynamic instability, and preoperative malnutrition, anemia, or intestinal obstruction.

### 2.2 Surgical procedure and perioperative management

All patients underwent elective laparoscopic radical colorectal resection (including laparoscopic radical colectomy or laparoscopic total mesorectal excision according to tumor location) performed by the same chief surgeon using standardized oncologic principles. Patients fasted for 6 hours and abstained from clear fluids for 2 hours preoperatively.

After entering the operating room, oxygen therapy was provided, intravenous access was established, and continuous electrocardiography and bispectral index (BIS) monitoring were initiated. General anesthesia was induced with midazolam (0.04 mg/kg), sufentanil (0.4–0.6 μg/kg), rocuronium (0.6 mg/kg), and propofol (1–2 mg/kg), followed by tracheal intubation and mechanical ventilation. Anesthesia was maintained using target-controlled infusions of propofol (3–4 μg/mL) and remifentanil (3–5 ng/mL), with supplemental sufentanil and rocuronium administered as needed. BIS was maintained between 40 and 60 with stable hemodynamics.

At the end of surgery, anesthetics were discontinued, patient-controlled intravenous analgesia (PCIA) was initiated, and patients were transferred to the recovery unit for extubation after regaining consciousness.

### 2.3 Group allocation

Participants were randomly assigned in a 1:1 ratio to the multimodal intervention (MI) group or the control group (n = 133 per group) using a computer-generated random number table with sealed opaque envelope concealment.

### 2.4 Routine postoperative care

Patients in the control group received standardized postoperative care. Vital signs (blood pressure, heart rate, respiratory rate, temperature, and oxygen saturation) were monitored every 4 hours during the early postoperative period. Analgesia consisted of oral acetaminophen 0.5–1.0 g every 6 hours as needed, with intravenous flurbiprofen axetil 50 mg every 12 hours or tramadol 50–100 mg every 12 hours administered when pain control was inadequate.

Postoperative nausea and vomiting (PONV) were treated with ondansetron 4 mg or metoclopramide 10 mg intravenously every 8 hours as required, with dexamethasone 5 mg added when clinically indicated. Intravenous fluid therapy was adjusted daily according to intake–output balance and laboratory results. Routine postoperative education and general psychological support were provided during daily ward rounds without structured behavioral intervention.

### 2.5 Multimodal intervention

In addition to routine care, the MI group received a structured multimodal program including optimized PCIA (sufentanil 1–2 μg/kg, tramadol 400 mg, and flurbiprofen axetil or parecoxib 200 mg diluted to 100 mL; background 2 mL/h, bolus 2 mL, lockout 15 min) maintained for 48–72 hours with rescue acetaminophen as needed; standardized PONV prophylaxis with ondansetron 4 mg or tropisetron 5 mg at the end of surgery; semi-recumbent positioning (30–45°) with repositioning every 2 hours; staged early oral intake beginning 6–12 hours postoperatively; protocolized early mobilization from 6 hours after surgery; early removal of nasogastric tubes (6–12 h), urinary catheters (24–48 h), and drains when appropriate; daily family companionship; structured psychological counseling (15–20 min/day) with Hospital Anxiety and Depression Scale screening; music therapy (60–80 bpm, 20–30 min twice daily); and circadian lighting management (daytime 200–500 lx, nighttime <50 lx) with optional melatonin 3 mg before sleep when necessary.

### 2.6 Outcome measures and instrument reliability

The primary outcome was total sleep time on postoperative night 6 (N6), and the secondary outcome was the Pittsburgh Sleep Quality Index (PSQI) score on postoperative day 7 (D7).

Objective sleep data were obtained using the Huawei WATCH 3 wearable device on the night 2 days before surgery (N-2), the operative night (N0), and postoperative night 6, recording total sleep time, sleep stages, and awakenings; prior studies have demonstrated acceptable agreement between validated wearable devices and polysomnography in clinical settings.

Subjective sleep quality and recovery were assessed using the PSQI and Quality of Recovery-15 (QoR-15) on D-1, D1, and D7. The Chinese versions of PSQI and QoR-15 have demonstrated good internal consistency and reliability in perioperative populations. Anxiety and depression were screened using the Hospital Anxiety and Depression Scale (HADS), and pain intensity was assessed using the Numeric Rating Scale (NRS). Perioperative complications and NRS scores on postoperative days 1–3 were recorded.

### 2.7 Sample size calculation

Sample size was calculated using PASS 15.0 based on pilot data showing a mean PSQI score of 17.0 ± 4.8 on postoperative day 1. Assuming a clinically meaningful reduction of 2.0 points, a two-sided α of 0.05, and power of 0.90, the minimum required sample size was 121 patients per group. Allowing for a 10% dropout rate, 133 patients were included in each group (total n = 266).

### 2.8 Statistical analysis

Statistical analyses were performed using SPSS version 23.0 (IBM Corp., Armonk, NY, USA). Normally distributed continuous variables are presented as mean ± standard deviation and compared using the independent-samples t-test, whereas non-normally distributed data are expressed as median (interquartile range) and analyzed using the Mann–Whitney U test. Categorical variables are reported as n (%) and compared using the chi-square test or Fisher’s exact test as appropriate. A two-sided P < 0.05 was considered statistically significant.

## 3 Results

### 3.1 Comparison of general information between the two groups

All patients in the two groups completed the study without any serious complications or withdrawal. There were no significant differences in gender, age, BMI, surgery time, anesthesia time, recovery time, and anesthetic dosage between the two groups (*p*>0.05, **Table 1**).

**Table 1:** Comparison of general information between two groups of patients (n=133).

Groups	Gender (male/female, number, %)	Age (year)	Body mass index (kg/m^2^)	Operative time (min)	Anesthesia duration (min)	Wake-up time (min)	Propofol dosage (mg)	Sufentanil dosage(μg)
Intervention Group	81(60.9)/52(39.1)	64.3 ± 7.6	24.2 ± 4.5	157.3 ± 31.6	203.7 ± 45.3	21.9 ± 5.7	1227.1 ± 145.6	69.5 ± 8.1
Control Group	79(59.4)/54(40.6)	62.7 ± 7.4	24.8 ± 4.9	152.6 ± 30.8	199.3 ± 43.6	21.2 ± 5.3	1246.2 ± 151.2	71.3 ± 9.2
Statistical value	0.622^a^	-0.568	0.437	-0.602	-0.382	-0.306	0.682	0.461
*P* value	0.529	0.596	0.723	0.559	0.747	0.812	0.519	0.712

^a^is expressed as an example (%), using the chi square test; The remaining data is expressed as mean ± SD and *t*-test is used.

### 3.2 Comparison of sleep quantity at different times between the two groups

**Figure 2:**
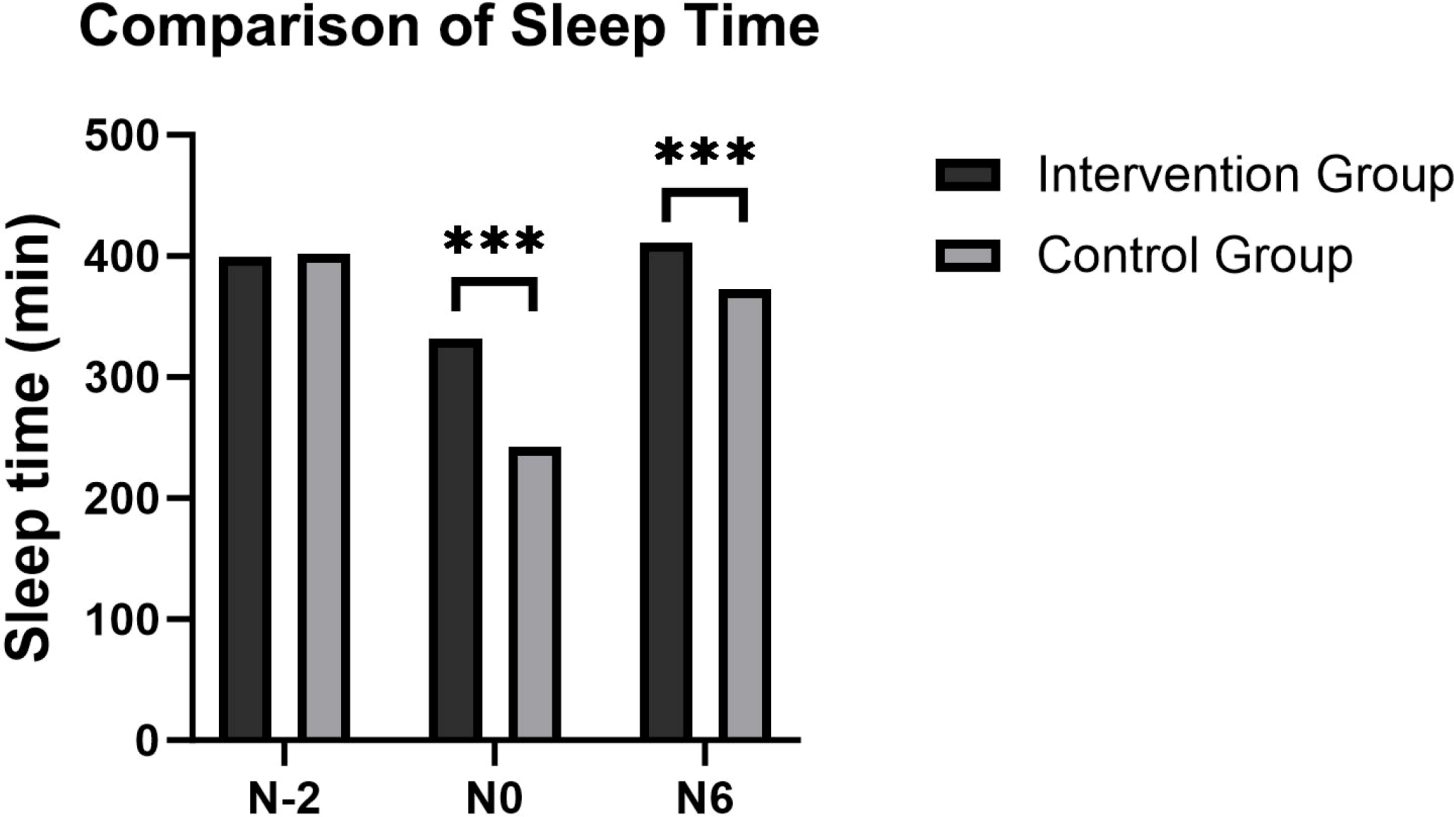
Comparison of primary outcome measure: postoperative sleep time. ***P<0.001.

There were no significant differences between the two groups in sleep latency, wake time after sleep onset, total sleep duration, or sleep efficiency on the second night before surgery (N-2) (**Figure 2**; p > 0.05). Compared with N-2, both groups exhibited prolonged sleep latency, increased wake time after sleep onset, reduced total sleep duration, and decreased sleep efficiency on the night of surgery (N0) (p < 0.05). By the sixth postoperative night (N6), patients in the control group continued to experience increased wake time, decreased sleep duration, and reduced sleep efficiency compared with N0 (p < 0.05). In contrast, the intervention group demonstrated significantly shorter sleep latency, reduced wake time, longer sleep duration, and higher sleep efficiency on both N0 and N6 compared with the control group (p < 0.05, **Table 2**).

**Table 2:** Comparison of sleep structure between two groups of patients at different times (*n* = 133).

Groups	Sleep time (min)			Sleep efficiency (%)			Sleep latency period (min)			Awakening time after falling asleep (min)		
	N_-2_	N_0_	N_6_	N_-2_	N_0_	N_6_	N_-2_	N_0_	N_6_	N_-2_	N_0_	N_6_
Intervention Group	399.2 ± 22.1	331.5 ± 30.7^ab^	411.6 ± 29.1^b^	83.2 ± 4.2	69.1 ± 3.7^ab^	85.8 ± 4.7^b^	24.5 ± 5.9	35.7 ± 7.2^ab^	22.3 ± 5.1	56.4 ± 8.2	112.3 ± 20.7^ab^	46.2 ± 7.1^b^
Control Group	402.1 ± 23.3	242.9 ± 24.3^a^	372.7 ± 25.9^a^	83.8 ± 4.3	50.6 ± 3.2^a^	77.6 ± 4.2^a^	23.1 ± 5.2	49.5 ± 8.2^a^	28.1 ± 5.8	54.9 ± 7.6	187.6 ± 27.2^a^	79.1 ± 9.5^a^
*t* value	0.417	-4.939	-4.513	0.207	-4.237	-2.786	-0.316	5.161	0.712	-0.682	4.062	3.216
*P* value	0.728	<0.001	<0.001	0.912	<0.001	0.029	0.792	<0.001	0.462	0.497	<0.001	0.005

According to *t*-test, compared with N_-2_, ^a^*P* < 0.05; Compared with Control Group, ^b^*P* < 0.05.

### 3.3 Comparison of PSQI scores and QoR-15 scores between the two groups at different time points

There were no significant differences in PSQI and QoR-15 scores between the two groups at the D_-1_ level (*p*>0.05). Compared with D_-1_, both groups showed an increase in PSQI scores and a decrease in QoR-15 scores on D_1_ and D_7_ (*p* < 0.05). Compared with control group, intervention group had lower PSQI and higher QoR-15 scores at D_1_ and D_7_ (*p* < 0.05, **Table 3**).

**Table 3:** Comparison of PSQI and SDS scores between two groups of patients at different time points (n=133).

Groups	PSQI scores			QoR-15 scores		
	D_-1_	D_1_	D_7_	D_-1_	D_1_	D_7_
Intervention Group	6.2 ± 2.4	14.2 ± 3.7^ab^	6.5 ± 3.1^ab^	137.9 ± 8.2	106.2 ± 7.5^ab^	122.8 ± 8.5^ab^
Control Group	5.9 ± 2.3	17.1 ± 4.1^a^	8.7 ± 3.8^a^	136.3 ± 8.1	93.8 ± 6.8^a^	109.5 ± 7.9^a^
*t* value	0.386	5.542	3.872	0.351	3.162	2.821
*P* value	0.798	<0.001	<0.001	0.825	0.003	0.039

According to *t*-test, compared with D_-1_, ^a^*P* < 0.05; Compared with Control Group, ^b^*P* < 0.05.

### 3.4 Comparison of NRS pain scores between the two groups at different postoperative time points

There were no significant differences in resting NRS pain scores and exercise NRS pain scores at D_3_ between the two groups (*p*>0.05). Compared with control group, intervention group had lower NRS pain scores during exercise at D_1_ and D_2_ (*p* < 0.05, **Table 4**).

**Table 4:** Comparison of NRS pain scores between two groups of patients at different postoperative time points (n=133, M (*P*25, *P*75)).

States	Groups	D_1_	D_2_	D_3_
Quiescent	Intervention Group	3.0(2.0,4.0)	2.0(2.0,3.0)	2.0(1.0,2.0)
	Control Group	4.0(3.0,5.0)	3.0(3.0,4.0)	3.0(2.0,3.0)
	*Z* value	0.473	0.524	0.295
	*P* value	0.642	0.601	0.819
Activity	Intervention Group	4.0(2.0,5.0)^b^	3.0(3.0,4.0)^b^	3.0(2.0,3.0)
	Control Group	6.0(4.0,7.0)	5.0(4.0,6.0)	4.0(3.0,5.0)
	*Z* value	2.615	2.348	0.582
	*p* value	0.025	0.031	0.592

According to the U-test, compared with Control Group, ^b^*P* < 0.05.

### 3.5 Comparison of postoperative adverse reactions and satisfaction between the two groups

Compared with control group, intervention group had a lower incidence of postoperative nausea and vomiting, more cases of rescue analgesia, and higher satisfaction (*P* < 0.05). There were no significant differences in other adverse reactions between the two groups (*p*>0.05, **Table 5**).

**Table 5:** Comparison of postoperative adverse reactions and satisfaction scores between two groups of patients (n=133).

Groups	Hypotension (number, %)	Bradycardia (number, %)	Nausea and vomiting (number, %)	Respiratory depression (number, %)	Dizzy (Number, %)	Remedial analgesia (number, %)	Satisfaction score
Intervention Group	2(1.50)	1(0.75)	21(15.8)^c^	0(0)	12(9.02)	53(39.8)^c^	8.8 ± 0.4^c^
Control Group	3(2.26)	3(2.26)	39(29.3)	1(0.75)	19(14.3)	12(9.02)	7.1 ± 0.3
Statistical value			2.826^b^		0.492^b^	4.76^b^	2.516^a^
*P* value	0.795	0.131	0.037	0.842	0.721	<0.001	0.043

^a^data is represented by mean ± SD, and *t*-test is used. ^b^data is presented as number (%) using χ^2^ test. ^c^data is presented using Fisher’s exact probability method.

## 4 Discussion

PSD is characterized by alterations in sleep architecture, including prolonged sleep latency, increased fragmentation, reduced rapid eye movement (REM) and slow-wave sleep, and overall decreased sleep duration and quality (15). PSD affects over 50% of surgical patients and is closely associated with delayed recovery, increased pain perception, and poorer prognosis (10, 16). Surgical stress, anesthetic agents, and perioperative physiological changes are major contributors to PSD, and early identification of high-risk patients is essential to optimize postoperative recovery (17). Pharmacological treatments for PSD are limited by potential adverse effects, including respiratory depression, highlighting the need for safe and effective non-pharmacological strategies.

MI, as a complementary approach within the biopsychosocial framework, can modulate neuroendocrine function, promote monoamine neurotransmitter release, and stimulate hippocampal neurogenesis, thereby improving both structural and functional aspects of the central nervous system that underlie sleep regulation (11, 12). Given that the effects of anesthesia and surgery on brain function are typically transient and reversible (18), MI is physiologically well-positioned to enhance perioperative sleep and recovery.

In clinical practice, resource limitations and staffing constraints may compromise postoperative care. An optimized MI program integrates continuous, real-time monitoring and active participation by patients and families, complementing routine postoperative management. Key elements include standardized analgesia, early mobilization, early enteral intake, prevention and treatment of nausea and vomiting (PONV), catheter and drain management, maintenance of comfortable positioning, psychological support, music therapy, and circadian-aligned lighting (19, 20). In our study, both groups received standardized ERAS-based perioperative care, while the MI group additionally received the enriched environment protocol. This approach allows us to attribute observed improvements primarily to the MI components while acknowledging that optimized multimodal perioperative management may collectively contribute to better outcomes.

Our findings demonstrate that patients undergoing laparoscopic radical colorectal surgery experienced PSD with decreased total sleep time, reduced sleep efficiency, prolonged sleep latency, and increased wake time, alongside elevated PSQI scores and decreased QoR-15 scores in the early postoperative period. Notably, the MI group exhibited faster recovery of sleep and functional outcomes to near preoperative levels, lower exercise-related NRS pain scores, reduced incidence of PONV, and higher satisfaction compared with the control group. These results support the clinical effectiveness and acceptability of MI as a non-pharmacological intervention to enhance postoperative sleep and recovery.

Several limitations should be acknowledged. First, this was a single-center study with a moderate sample size, which may limit external validity. Second, while randomization was implemented, the open-label design inherent to environmental and behavioral interventions could introduce performance and assessment bias. Third, although wearable sleep monitors are validated and practical, they do not capture the full detail of sleep architecture compared with polysomnography. Fourth, the follow-up period was limited to the early postoperative phase, leaving the long-term sustainability of benefits unclear. Finally, MI was applied as a bundled intervention, making it challenging to isolate the effects of individual components. Additionally, variations in analgesia, PONV management, and catheter/drain removal—although standardized and adjusted in analysis—may partially contribute to observed differences. Future multicenter trials with larger cohorts, extended follow-up, objective sleep validation, and component-specific analyses are warranted to confirm and refine these findings.

## 5 Conclusion

In conclusion, among patients undergoing laparoscopic radical resection for CRC, an MI can improve postoperative sleep quality and recovery quality, and promote postoperative recovery.

## Data Availability

The original contributions presented in the study are included in the article/supplementary material. Further inquiries can be directed to the corresponding authors.
